# Prognostic significance of circulating tumor cells in non-small cell lung cancer patients undergoing chemotherapy

**DOI:** 10.18632/oncotarget.21255

**Published:** 2017-09-23

**Authors:** Bing Tong, Yan Xu, Jing Zhao, Minjiang Chen, Jia Xing, Wei Zhong, Mengzhao Wang

**Affiliations:** ^1^ Department of Respiratory Medicine, Lung Cancer Center, Peking Union Medical College Hospital, Peking Union Medical College, Chinese Academy of Medical Sciences, Beijing, 100730 P.R. China; ^2^ Cyttel Biosciences Inc., Beijing, 101111 P.R. China

**Keywords:** circulating tumor cell, non-small cell lung cancer, biomarker, prognosis, chemotherapeutic response

## Abstract

The utility of circulating tumor cells (CTCs) as prognostic biomarkers in non-small cell lung cancer (NSCLC) is inconclusive due to the limitations of current CTC detection methods. Using a novel high-efficiency detection method, we determined the ability of CTCs to predict survival and chemotherapeutic responses in NSCLC. In 127 patients with advanced NSCLC, CTCs were counted and analyzed at baseline and during follow-up. Median overall survival (OS) and progression-free survival (PFS) were longer in patients with baseline CTC counts <8 CTCs/3.2 mL (20.0 vs. 10.4 months [P = 0.009] and 7.2 vs. 5.5 months [P < 0.001], respectively). Patients with post-treatment increases in the CTC count had poorer OS and PFS than those without increases (12.0 vs. 13.3 months [P = 0.028] and 5.2 vs. 6.4 months [P = 0.022], respectively). There was no association between the baseline CTC count and chemotherapeutic response (P = 0.734). However, the rate of progressive disease in patients with and without post-treatment increases in the CTC count were 15.6% and 2.4% (P = 0.042), respectively. The baseline CTC count and the change in the CTC count during treatment were both valuable prognostic indicators for NSCLC.

## INTRODUCTION

In recent years, considerable efforts have been made toward the identification of prognostic and predictive biomarkers to guide personalized medicine approaches for patients with advanced non-small cell lung cancer (NSCLC). Circulating tumor cells (CTCs) have been established as a prognostic and predictive biomarker for metastatic breast, colorectal and prostate cancers, with growing evidence suggesting a similar role in lung cancer [1–5]. In a preliminary study [6], we demonstrated the potential of the baseline CTC count as an independent negative prognostic factor for advanced NSCLC. However, there was no statistically significant association between the change in the CTC count and survival outcomes or treatment response. The aim of this study was to further validate the clinical significance of the baseline CTC count and the change in the CTC count during treatment with standard chemotherapy to predict survival outcomes and chemotherapeutic response in patients with advanced NSCLC.

## RESULTS

### Patient characteristics

**Figure 1:**
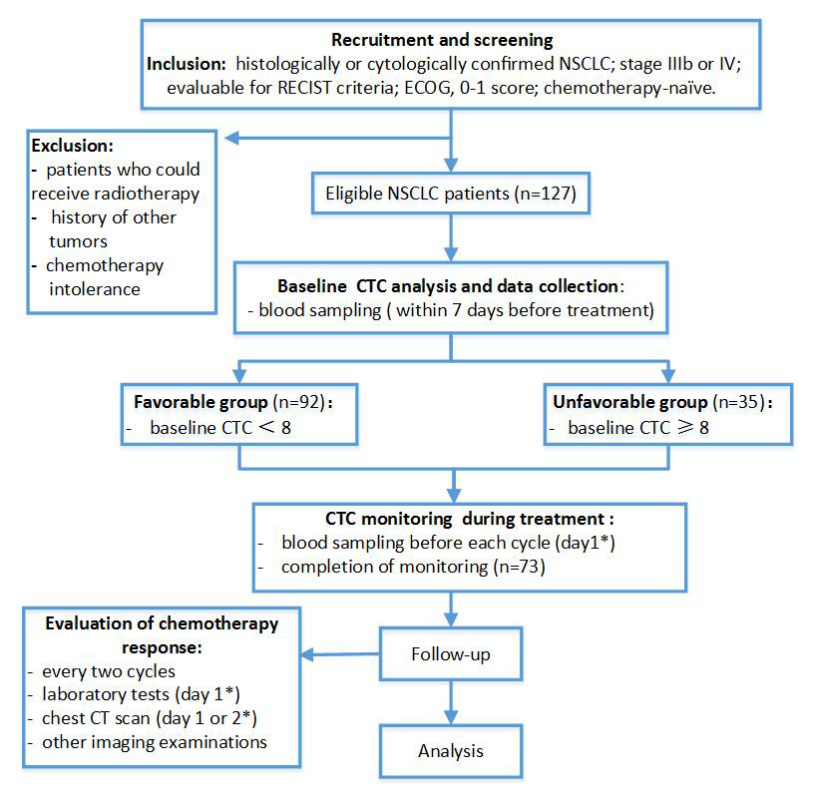
Flow chart of study design * From the first day of hospitalization.

Between September 2012 and September 2015, 127 patients met the inclusion criteria and were included in the primary analysis. Among them, only 73 had their CTCs analyzed before the first, second and third cycle of chemotherapy (Figure 1). Patient characteristics at the point of study entry are summarized in Table 1. Of the 89 lung adenocarcinoma patients, 72 underwent testing for epidermal growth factor receptor (EGFR) mutation and anaplastic lymphoma kinase (ALK) rearrangement. The remaining 17 lung adenocarcinoma patients did not undergo testing because of insufficient tumor tissue. Using the amplification-refractory mutation system, EGFR mutations were detected in 25 patients (L858R point mutations [n = 7] and exon 19 deletions [n = 18]). Using the Ventana immunohistochemistry platform, only 3 patients were identified as having ALK rearrangements. However, all patients who underwent mutational analyses had already undergone standard first-line chemotherapy before the test results were obtained. Eventually, of the 127 patients, 50 were treated with pemetrexed plus cisplatin, 30 with docetaxel plus cisplatin, 29 with gemcitabine plus cisplatin and 18 with paclitaxel plus carboplatin. At the time of the final analysis, 7 patients in the favorable group and 2 patients in the unfavorable group had undergone targeted therapy after the disease had progressed.

**Table 1:** Patient demographics and clinical characteristics

Characteristics	n=127	%
Age at baseline, yrs		
Median	59	
Range	33-78	
Gender		
Male	82	64.6
Female	45	35.4
Current or former smokers	80	63.0
ECOG		
0	102	80.3
1	25	19.7
Histology		
ADC	90	70.9
SCC	37	29.1
Tumor stage		
Stage IIIb	21	16.5
Stage IV	106	83.5
EGFR mut		
Yes	25	19.7
No	62	48.8
Unknown	40	31.5
Metastasis location		
Bone	50	39.4
Liver	12	9.4
Adrenal gland	12	9.4
Brain	11	8.7
Distant lymphnode*	13	10.2
Others**	5	4.0

ADC, adenocarcinoma; SCC, squamous cell carcinoma.

Distant lymphnode*: Supraclavicular lymph nodes (n=8), axillary lymph nodes (n=3) and celiac lymph nodes (n=2).

Others**: Pericardium (n=2), kidney (n=1), meninges (n=1), and pancreas (n=1).

### CTCs at baseline and during follow-up

**Figure 2:**
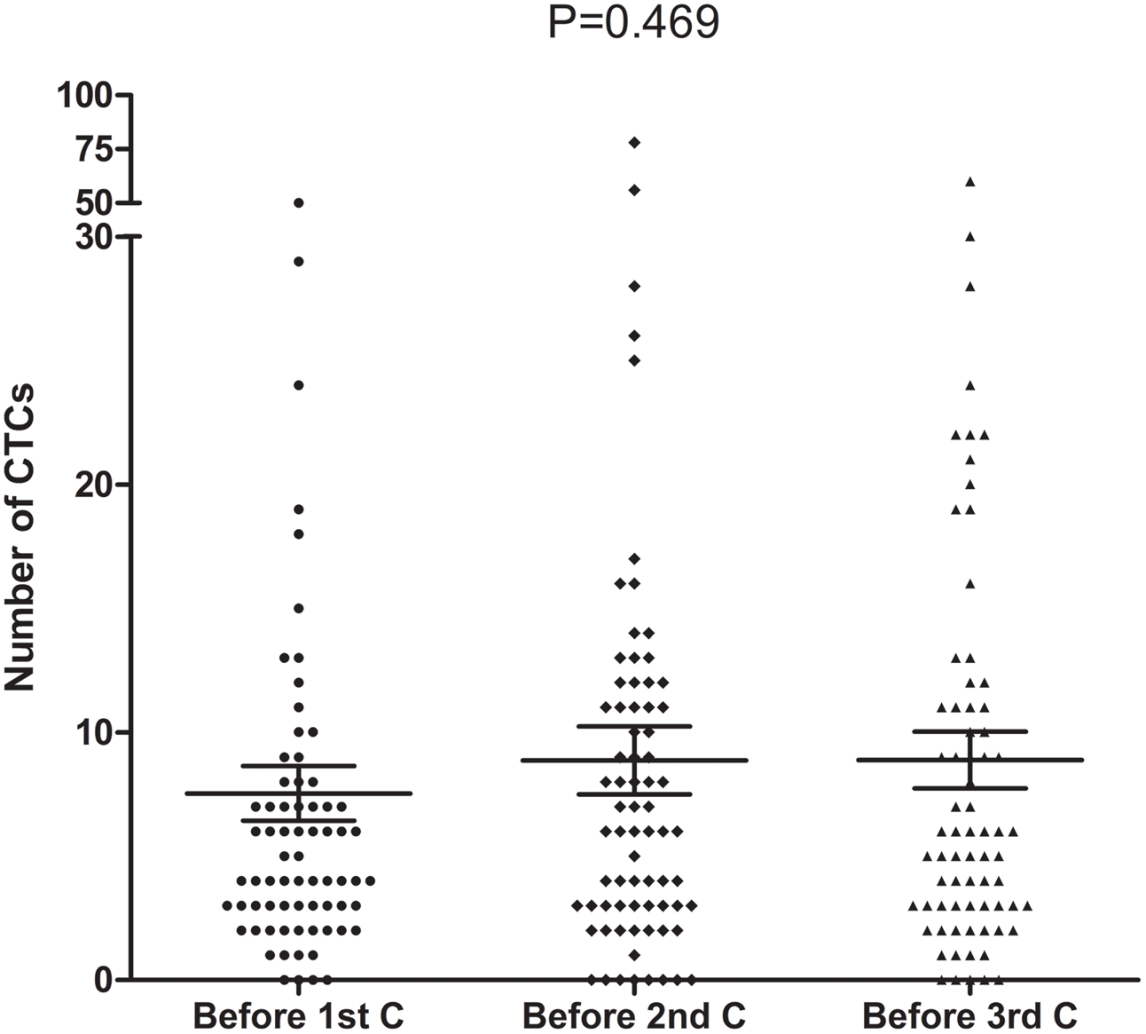
CTC counts before the first, second and third cycle of chemotherapy (n=73, P=0. 469)

In total, 107 patients were positive for ≥2 CTCs/3.2 mL of blood at baseline: 90, ≥3; 71, ≥4; 56, ≥5; 53, ≥6; 43, ≥7; 35, ≥8. There was no significant correlation between the favorable or unfavorable baseline CTC count and patient clinicopathological characteristics (Table 2). Among the 73 patients that had their CTCs analyzed before all three cycles of chemotherapy, the median CTC count was 4 (range, 0–80), 6 (range, 0–78) and 2 (range, 0–60) CTCs/3.2 mL of blood before the first, second and third cycle of chemotherapy, respectively. There was no significant difference between them (P = 0.469, Figure 2). After 2 cycles of chemotherapy, 35 patients showed a decrease in the CTC count, 32 patients showed an increase in the CTC count and 6 patients showed no change in the CTC count.

**Table 2:** Characteristics of the favorable (CTC < 8) and unfavorable (CTC ≥ 8) groups

Characteristics	Total (n = 127)	Baseline CTC count		P value
		< 8 (n = 92)	≥ 8 (n = 35)	
Smoking history, n (%)				
Yes	80 (63.0)	59 (73.8)	21 (26.2)	
No	47 (37.0)	33 (70.2)	14 (29.8)	0.667
Histology, n (%)				
ADC	89 (70.1)	63 (70.8)	26 (29.2)	
SCC	38 (29.9)	29 (76.3)	9 (23.7)	0.523
Tumor stage, n (%)				
IIIb	21 (16.5)	17 (81.0)	4 (19.0)	
IV	106 (83.5)	75 (70.8)	31 (29.2)	0.339
EGFR mutation, n (%)				
Positive	25 (19.7)	20 (80.0)	5 (20.0)	
Negative	62 (48.8)	42 (67.7)	20 (32.3)	
Unknown	40 (31.5)	30 (75.0)	10 (25.0)	0.465
Metastatic site, n (%)				
Bone				
Yes	50 (39.4)	38 (76.0)	12 (24.0)	
No	77 (60.6)	54 (70.1)	23 (29.9)	0.469
Liver				
Yes	12 (9.4)	9 (75.0)	3 (25.0)	
No	115 (90.6)	83 (72.2)	32 (27.8)	1.000*
Adrenal gland				
Yes	12 (9.4)	10 (83.3)	2 (16.7)	
No	115 (90.6)	82 (71.3)	33 (28.7)	0.584*
Brain				
Yes	11 (8.7)	8 (72.7)	3 (27.3)	
No	116 (91.3)	84 (72.4)	32 (27.6)	1.000*
Distant lymph nodes				
Yes	13 (10.2)	10 (76.9)	3 (23.1)	
No	114 (89.8)	82 (71.9)	32 (28.1)	0.957*
No. of distant metastases, n (%)				
0	21 (16.5)	17 (81.0)	4 (19.0)	
1	63 (49.6)	43 (68.3)	20 (31.7)	
≥ 2	43 (33.9)	32 (74.4)	11 (25.6)	0.497

*Continuity correction

### Prognostic significance of CTCs

**Figure 3:**
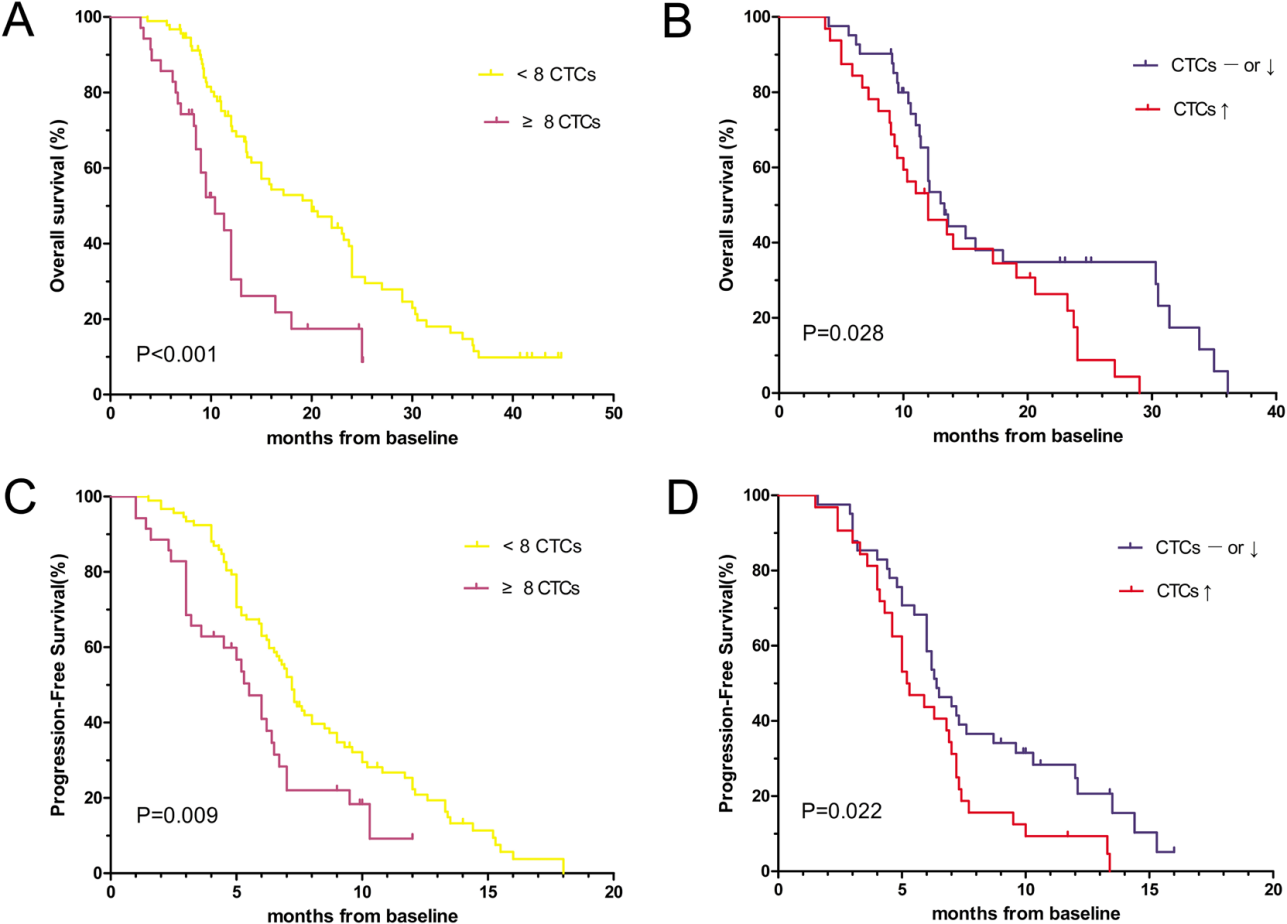
Kaplan-Meier curves of **(A)** OS according to the baseline CTC count (n=127), **(B)** OS according to the post-treatment CTC count (n=73), **(C)** PFS according to the baseline CTC count (n=127) and **(D)** PFS according to the post-treatment CTC count (n=73) in patients with advanced (Stage IIIB or Stage IV) NSCLC.

At the time of the last follow-up (July 30, 2016), 108 of 127 patients experienced disease progression, and 91 patients died. The median follow-up duration of the 36 surviving patients was 10.3 months (range, 8.7–44.8 mo). Median OS time was significantly longer in the favorable group versus the unfavorable group (20.2 vs. 10.4 mo, respectively; log-rank test, P < 0.001; Figure 3A). An unfavorable baseline CTC count and a smoking history were associated with poorer OS, as indicated by step-wise multivariate analysis (Table 3, Supplementary Table 1). The mortality risk was significantly higher for smokers (hazard ratio, 1.680; 95.0% confidence interval, 1.049-2.695; P = 0.031) and significantly lower for patients with a favorable baseline CTC count (hazard ratio, 0.437; 95.0% confidence interval, 0.268-0.713; P = 0.001). There was not a significant association between survival outcomes and other clinical factors, including the Eastern Cooperative Oncology Group (ECOG) performance status (PS), EGFR mutation status, metastasis, age, sex, histology and tumor stage (Supplementary Table 1). Moreover, among the 73 patients that had their CTCs analyzed before all three cycles of chemotherapy, patients with post-treatment increases in the CTC count (n = 32) had poorer median OS than those with no changes or post-treatment decreases (n = 41; 12.0 vs. 13.3 mo, P = 0.028; Figure 3B).

**Table 3:** Univariate and multivariate Cox proportional hazards regression analysis for prediction of PFS and OS

Variable	OS		PFS	
	HR (95.0% CI)	P value	HR (95.0% CI)	P value
Univariate Cox proportional hazards regression analysis				
Age:<60 vs. ≥60	0.672 (0.443-1.021)	0.063	0.906 (0.618-1.326)	0.611
Sex: female vs. male	0.639 (0.406-1.007)	0.054	0.894 (0.598-1.337)	0.586
Smoking history: yes vs. no	1.836 (1.152-2.926)	**0.011**	1.312 (0.879-1.960)	0.184
ECOG PS: 0 vs. 1	0.828 (0.496-1.381)	0.469	0.798 (0.489-1.304)	0.368
Histology: SCC vs. ADC	0.713 (0.459-1.109)	0.134	0.914 (0.606-1.377)	0.666
EGFR mutation: unknown vs. no vs. yes	1.085 (0.832-1.415)	0.546	1.281 (0.986-1.644)	0.064
Tumor stage: IIIb vs. IV	0.974 (0.550-1.725)	0.927	0.798 (0.474-1.344)	0.397
Distant metastases: yes vs. no	1.084 (0.611-1.923)	0.783	1.348 (0.806-2.254)	0.255
Baseline CTCs: <8 vs. ≥8	0.401 (0.246-0.654)	**<0.001**	0.561 (0.359-0.875)	0.011
Stepwise multivariate Cox proportional hazards regression analysis				
Baseline CTCs: <8 vs. ≥8	0.437 (0.268-0.713)	**0.001**	0.561 (0.359-0.875)	**0.011**
Smoking history: yes vs. no	1.680 (1.049-2.695)	**0.031**	—	0.205

Furthermore, there was a significant difference in median PFS between the favorable and unfavorable groups (7.2 vs. 5.5 mo; log-rank test, P = 0.009; Figure 3C). In univariate and multivariate analyses, the baseline CTC count was significantly associated with PFS, demonstrating a lower risk of disease progression in the favorable group (hazard ratio, 0.561; 95.0% confidence interval, 0.359-0.875; P = 0.011; Table 3, Supplementary Table 1). In addition, PFS was shorter in patients (n = 32) with post-treatment increases in the CTC count than in patients (n = 41) without post-treatment increases in the CTC count (5.2 vs. 6.4 mo, P = 0.022; Figure 3D).

### CTCs and chemotherapeutic responses

**Figure 4:**
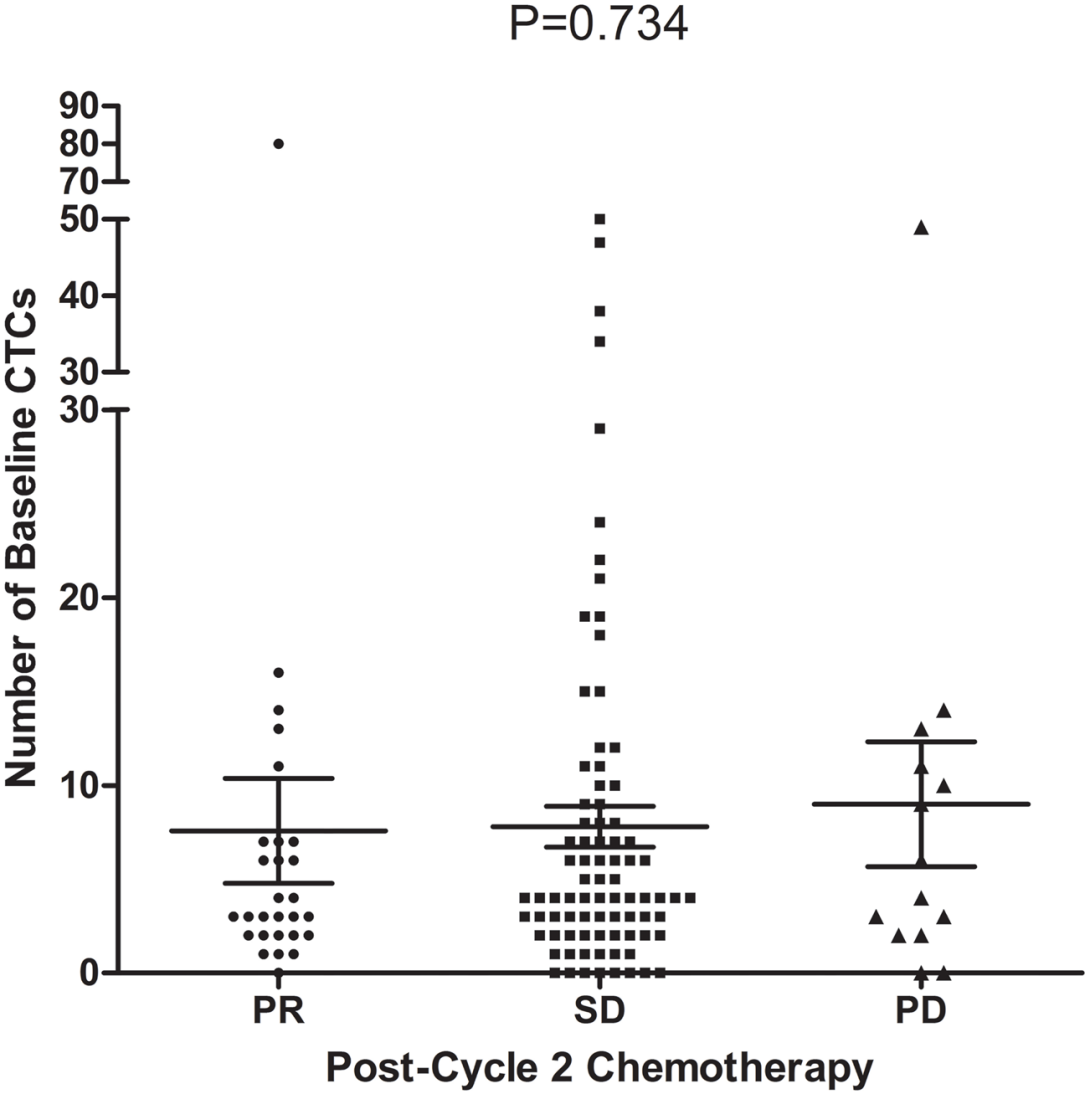
Relationship between the baseline CTC count and chemotherapeutic response (n=125, P=0. 734)

Of the 127 patients enrolled, 125 patients underwent tumor assessments, with 2 patients lost to follow-up after the second cycle of chemotherapy. A partial response (PR) was observed in 28 patients, stable disease (SD) was observed in 83 and progressive disease (PD) was observed in 14. There was no significant difference in the baseline CTC count between the three groups (3.0 vs. 4.0 vs. 5.0; P = 0.734; Figure 4; Table 4), and there was no significant difference in the chemotherapeutic response between the favorable and unfavorable groups (Table 4).

**Table 4:** Association between CTC count and treatment response in the chemotherapy group

Parameter	PR	SD	PD
Baseline CTC count			
Median	3	4	5
<8 (n=90)	25.6%(23)	65.6%(59)	8.9%(8)
≥8 (n=35)	14.3%(5)	68.6%(24)	17.1%(6)
P-value	0.175	0.749	0.189
Change in CTC count after 2 cycles of chemotherapy			
Unchanged/decreased (n=41)	26.8%(11)	70.7%(29)	2.4%(1)
Increased (n=32)	18.8%(6)	65.6%(21)	15.6%(5)
P-value	0.418	0.641	**0.042**

PD, progressive disease; PR, partial response; SD, stable disease.

Among the 73 patients that had their CTCs analyzed before all three cycles of chemotherapy, 17 exhibited a PR, 50 had SD and 6 had PD. Interestingly, the persistence of a high CTC count was associated with poor response to chemotherapy (Table 4), as PD rates were significantly different between patients with post-treatment increases in the CTC count and patients without post-treatment increases in the CTC count (15.6% vs. 2.4%, respectively, P = 0.042).

## DISCUSSION

Multiple studies have investigated CTCs and their prognostic role in NSCLC and other cancer types [2, 6–8]. Here, we confirmed that the baseline CTC count and the change in the CTC count during chemotherapy serve as strong independent predictors of OS and PFS in patients with NSCLC.

In this study, CTC detection was performed using the Cyttel method, which combines anti-CD45 immunohistochemistry and fluorescence *in situ* hybridization. Our detection rate was considerably higher than those obtained using the CellSearch system. The CellSearch system revealed that only 30–50% of lung cancer patients had ≥1 CTC/7.5 mL of blood, and just 20–30% of patients had ≥2 CTCs/7.5 mL of blood [9–11]. Discrepancies between CTC detection rates might arise as a consequence of using different enrichment strategies.

Epithelial cell adhesion molecule (EpCAM) is expressed at relatively low levels in patients with lung cancer [12]. The widely used CellSearch system and CTC-chip-based platforms detect CTCs via EpCAM and are, thus, inadequate at detecting CTCs in NSCLC. In contrast, the Cyttel method is an immunomagnetic bead-based CTC enrichment system that utilizes a leukocyte depletion mechanism, which allows detection of CTCs via an EpCAM-independent mechanism.

Strong evidence has emerged to support the utility of CTC counts as a prognostic indicator in NSCLC. Consistent with these studies [3, 13–15], our data show that survival rate is significantly poorer in patients with an unfavorable baseline CTC count. Change in the CTC count after chemotherapy was also correlated with survival. Patients with post-treatment increases in the CTC count had poorer clinical outcomes versus those without post-treatment increases. Similar observations have been reported in patients with breast, ovarian and prostate cancers [16–18].

In our study, 8 CTCs/3.2 mL of blood was identified as the most suitable CTC threshold versus 3 or 5 CTCs/3.2 mL of blood, which was previously reported [1–3]. Notably, the majority of previous studies using lower CTC thresholds were conducted using EpCAM-dependent methods. We postulate that this may have arisen from differences in sensitivity, reproducibility and specificity of different CTC detection techniques. Based on our findings, we recommend a threshold of 8 CTCs/3.2 mL of blood for defining favorable and unfavorable prognostic groups, especially for EpCAM-independent detection methods.

The present study demonstrated that post-treatment increases in the CTC count were significantly correlated with the rate of PD. Cohen et al. [1] showed that changes in the CTC count were associated with a meaningful sensitivity for predicting PD in colorectal cancer, which is consistent with our findings in NSCLC patients. Of note, we did not detect a relationship between the baseline CTC count and therapeutic response, which is consistent with the findings of our preliminary study [6]. Naito et al. [7] enrolled 51 treatment-naïve small cell lung cancer patients and also did not detect any significant association between therapeutic response and the baseline CTC count when using the CellSearch system. We found that patients with a favorable baseline CTC count tended to have higher PR rates and lower PD rates, however, these findings were not statistically significant. Larger, prospective, multicenter studies are needed to more fully clarify the association between the baseline CTC count and therapeutic response.

Our previous preliminary study found no association between the baseline CTC count and histological type, tumor stage, EGFR mutation status, metastasis site or the number of metastases [6]. In the present study, we also failed to detect any correlation between the CTC count and the above clinical characteristics, which disagrees with a previous report that suggested a higher CTC count was associated with an adenocarcinoma subtype and a greater number of metastatic sites [19]. These contradictory findings are likely due to small sample sizes and the presumption that specific CTC subtypes are important in tumor metastasis.

The main limitations of the present study are the limited proportion of patients who underwent consecutive CTC detection during chemotherapy (73 of 127 patients) and the lack of analysis of patients undergoing targeted therapy as first-line treatment. Moreover, it is difficult to compare our findings with those of other laboratories that used different detection techniques. However, additional data that could address these issues are expected from future, larger-scale, prospective, multicenter studies.

Overall, these findings demonstrate that the baseline CTC count and the change in the CTC count after chemotherapy are of significant utility in monitoring therapeutic response and predicting prognosis in patients with advanced NSCLC.

## MATERIALS AND METHODS

### Study design

This study was approved by the Ethics Committee of the Peking Union Medical College Hospital (Beijing, China). Informed written consent was obtained from each patient before participation in the study. Between September 2012 and September 2015, 127 consecutive treatment-naive patients with histologically proven advanced (Stage IIIb or Stage IV) NSCLC were included. Inclusion and exclusion criteria were applied as described previously [6] (Figure 1). Patients were enrolled at the Peking Union Medical College Hospital (Beijing, China) and later received standard first-line platinum-based chemotherapy. Peripheral blood samples (3.2 mL) were collected for CTC analysis at baseline (within 7 days before starting chemotherapy). Consistent with previous reports, 1 CTC/3.2 mL of blood was classified as CTC-negative, considering the potential for false-positive results, and ≥2 CTCs/3.2 mL of blood as CTC-positive [20–22]. Based on our preliminary work, a cut-off threshold of 8 CTCs/3.2 mL of blood was selected for stratifying patients into the favorable (<8 CTCs/3.2 mL of blood) and unfavorable (≥8 CTCs/3.2 mL of blood) prognostic groups [6]. We evaluated the relationships between the baseline CTC count and OS, PFS and treatment responses. Similar analyses were conducted for the CTC count change after 2 cycles of chemotherapy. Chemotherapeutic response was evaluated according to the Response Evaluation Criteria in Solid Tumors (version 1.1). OS was measured from the date of informed consent to the date of death or last follow-up. PFS was measured from the date of informed consent to the date of disease progression or death.

### Analysis of CTCs

CTCs were identified and counted using the Cyttel method, which combines subtraction enrichment, CD45 immunostaining and fluorescence *in situ* hybridization (FISH) [21]. The technical details, including the accuracy, linearity, and reproducibility of the method are described in our preliminary study [6]. Briefly, Samples were washed with CS1 buffer (Cyttel Biosciences Co., Ltd., Beijing, China), centrifuged at 650 g for 5 min to deplete the serum and processed by lysis of red blood cells with CS2 (Cyttel Biosciences Co., Ltd., Beijing, China). The residual components were re-suspended in CS1 buffer and incubated with immunomagnetic beads conjugated to anti-CD45 monoclonal antibody in order to separate white blood cells. CTCs were detected via CD45 immunostaining and FISH using anti-human CD45 and probes against the centromere of chromosome 8. CD45-negative, centromere of chromosome 8-positive, and 4’,6-diamidino-2-phenylindole-positive samples were considered CTC-positive.

### Statistical analyses

The primary analysis of this study was survival and its correlation to favorable and unfavorable baseline CTC counts. From previous studies, we predicted 1-year survival rates of patients with favorable and unfavorable baseline CTC counts to be 60% and 30%, respectively. Thus, a minimum sample size of 119 patients, including 90 patients with a favorable baseline CTC count and 29 with an unfavorable baseline CTC count, was required to achieve a statistical power of 0.90, with a two-sided log-rank test at the 5.0% significance level. In total, 127 eligible patients were enrolled in this study.

Differences amongst groups were analyzed using the Chi-squared test. Clinical factors that were significant predictors of OS and PFS in the univariate analysis were included in the forward stepwise multivariate Cox regression analysis (Wald method). Survival curves were plotted using the Kaplan-Meier method and compared using the log-rank test. All statistical analyses were conducted using Statistical Package for the Social Sciences for Windows (version 17.0) and GraphPad Prism (version 5.0). A P value of <0.05 was considered statistically significant.

## SUPPLEMENTARY MATERIALS TABLE



## Abbreviations

ADC: adenocarcinoma
ALK: anaplastic lymphoma kinase
CD45: leukocyte common antigen
CI: confidence interval
CTC: circulating tumor cell
ECOG: Eastern Cooperative Oncology Group
EGFR: epidermal growth factor receptor
EpCAM: epithelial cell adhesion molecule
NSCLC: non-small cell lung cancer
OS: overall survival
PD: progressive disease
PFS: progression-free survival
PR: partial response
PS: performance status
SCC: squamous cell carcinoma
SD: stable disease
