# Supplementary material for: Prognostic significance of circulating tumor cells in non-small cell lung cancer patients undergoing chemotherapy
Source: Oncotarget. 2017 Sep 23;8(49):86615–24. doi: 10.18632/oncotarget.21255 (PMC5689711; doi:10.18632/oncotarget.21255)
Supplement: Supplementary file 1 [file oncotarget-08-86615-s001.pdf]

Prognostic significance of circulating tumor cells in non-small cell lung cancer patients undergoing chemotherapy

SUPPLEMENTARY MATERIALS

Supplementary Table 1: Stepwise multivariate Cox analysis of overall survival (OS) and progression-free survival (PFS)

Variable	OS		PFS	
	HR (95.0% CI)	P value	HR (95.0% CI)	P value
Age:<60 vs. ≥60	—	0.359	—	0.960
Sex: female vs. male	—	0.748	—	0.582
Smoking history: yes vs. no	1.680 (1.049-2.695)	0.031	—	0.205
ECOG PS: 0 vs. 1	—	0.853	—	0.496
Histology: SCC vs. ADC	—	0.384	—	0.626
EGFR mutation: unknown vs. no vs. yes				
EGFR	—	0.426	—	0.199
EGFR (1)	—	0.208	—	0.178
EGFR (2)	—	0.509	—	0.884
Tumor stage: IIIb vs. IV	—	0.788	—	0.424
Distant metastases: yes vs. no	—	0.526	—	0.262
Baseline CTCs: <8 vs. ≥8	0.437 (0.268-0.713)	0.031	0.561 (0.359-0.875)	0.011

OS, overall survival; PFS, progression-free survival; CI, confidence interval; CTC, circulating tumor cell; EGFR, epidermal growth factor receptor; ECOG, Eastern Cooperative Oncology Group; ADC, adenocarcinoma; SCC, squamous cell carcinoma.
